# Can Ketogenic Diet Therapy Improve Migraine Frequency, Severity and Duration?

**DOI:** 10.3390/healthcare9091105

**Published:** 2021-08-26

**Authors:** Rebecca L. Haslam, Aaron Bezzina, Jaimee Herbert, Neil Spratt, Megan E. Rollo, Clare E. Collins

**Affiliations:** 1 Priority Research Centre for Physical Activity and Nutrition, The University of Newcastle, Callaghan, NSW 2308, Australia; rebecca.williams@newcastle.edu.au (R.L.H.); aaron.bezzina@uon.edu.au (A.B.); Megan.rollo@newcastle.edu.au (M.E.R.); 2 School of Health Sciences, College of Health Medicine and Wellbeing, The University of Newcastle, Callaghan, NSW 2308, Australia; jaimee.herbert@newcastle.edu.au; 3 Hunter Medical Research Institute, Kookaburra Circuit, New Lambton Heights, NSW 2305, Australia; 4 School of Biomedical Sciences and Pharmacy, College of Health, Medicine and Wellbeing, The University of Newcastle, Callaghan, NSW 2308, Australia; neil.spratt@newcastle.edu.au; 5 Department of Neurology, John Hunter Hospital, Hunter New England Local Health District, New Lambton Heights, NSW 2305, Australia

**Keywords:** migraine, ketogenic diet, anti-migraine diet

## Abstract

Migraine is the third most common condition worldwide and is responsible for a major clinical and economic burden. The current pilot trial investigated whether ketogenic diet therapy (KDT) is superior to an evidence-informed healthy “anti-headache” dietary pattern (AHD) in improving migraine frequency, severity and duration. A 12-week randomised controlled crossover trial consisting of the two dietary intervention periods was undertaken. Eligible participants were those with a history of migraines and who had regularly experienced episodes of moderate or mildly intense headache in the previous 4 weeks. Migraine frequency, duration and severity were assessed via self-report in the Migraine Buddy© app. Participants were asked to measure urinary ketones and side effects throughout the KDT. Twenty-six participants were enrolled, and 16 participants completed all sessions. Eleven participants completed a symptom checklist; all reported side-effects during KDT, with the most frequently reported side effect being fatigue (*n* = 11). All completers experienced migraine during AHD, with 14/16 experiencing migraine during KDT. Differences in migraine frequency, severity or duration between dietary intervention groups were not statistically significant. However, a clinically important trend toward lower migraine duration on KDT was noted. Further research in this area is warranted, with strategies to lower participant burden and promote adherence and retention.

## 1. Introduction

Migraine is the third most common condition worldwide and the sixth leading cause of disability, measured as years lived with disability (YLD) [1]. The condition is responsible for a major clinical and economic burden to individuals and societies, contributing to increased YLDs, decreased quality of life (QoL), lost productivity and increased healthcare and medications use [1]. Migraine is largely accepted as a primary disorder of brain excitatory–inhibitory balance where excessive neuronal activity activates the trigeminovascular pain pathway, resulting in central sensitisation and pain [2]. Acute and preventive therapies for migraine can be ineffective or poorly tolerated, highlighting a need for new pharmacological and non-pharmacological treatment strategies [3,4]. Dietetic intervention—in particular, the use of a very low-carbohydrate, high-fat ketogenic diet—has been considered theoretically in terms of a potential contribution to non-pharmacological treatment strategies for migraine.

The ketogenic diet was originally created for last-line medical nutrition therapy trials in refractory epilepsy [5]. The classic ketogenic diet is characterised by a high-fat (90%), low-carbohydrate and moderate-protein intake, sufficient to induce ketosis, whereby plasma concentrations of ketone bodies, β-hydroxybutyrate and acetoacetate are elevated [6]. A more classic ketogenic diet is one composed of a 4:1 ratio (4 g dietary fat to every 1 g combined protein plus carbohydrate); however, modified versions of the ketogenic diet have used ratios as low as 1:1 [7]. Trace amounts of total urinary ketones are seen at concentrations from 5 mg/dL (0.5 mmol/L). However, concentrations reported to elicit physiological benefits have been reported as needing to be as high as 80–160 mg/dL (13.8–27.5 mmol/L) [8]. During ketosis, these ketone bodies are used as the major energy source for all cells, including brain cells [6]. Elevated ketone body concentrations have exhibited anti-convulsive and neuroprotective properties in both in vitro and in vivo experimental studies [5,7,9]. These properties include ketone-induced changes in neurotransmitter balance, changes in neural membrane polarity to dampen elevated neuronal excitability associated with seizures and improved mitochondrial function due to increased energy reserves combined with decreased production of reactive oxygen species (ROS) [5,7,9].

Such metabolic changes are hypothesised to contribute to the positive clinical effects that have been observed in seizure activity in some patients with intractable epilepsy [9]. These effects, including a reduction in epileptic seizure activity by over 50% in both children and adults, have been documented in a number of clinical studies and systematic reviews over the past 20 years [7,9]. The benefits of a ketogenic diet for intractable epilepsy were also reported in a systematic review, where over 50% of children following a 4:1 ketogenic diet were seizure free, and 85% had a reduction in seizures after three months [10]. Given these clinical effects, the ketogenic diet is a therapy that should also be further evaluated for clinical efficacy in other neurological disorders, including but not limited to migraines [9].

There is only preliminary evidence to support efficacy of ketogenic diet therapy (KDT) as a medical nutrition therapy for treating migraine [2]. Preclinical studies in rats and mice suggest that KDT may positively impact specific stages of migraine pathophysiology, particularly in reducing propagation of cortical spreading depression (CSD), the neurophysiological event underpinning migraine aura [11]; protecting against neuro-inflammation [12]; reducing oxidative stress [13]; and enhancing brain metabolism [14]. Currently, very limited high-methodological-quality human clinical trials have been conducted. Di Lorenzo et al. recently conducted an RCT in overweight and obese migraine sufferers, comparing 1-month KDT to a very low calorie diet (VLCD); after 1-month on a KDT, there was a significant reduction in frequency and duration of migraines on the KDT compared to the VLCD group [15]. A 2017 narrative review summarised findings from case reports, prospective studies and RCTs (case reports *n* = 3, case series *n* = 1, RCT *n* = 2) that had investigated the effect of KDTs in treatment for migraine with and without aura, chronic headache and medication overuse headache [2]. The review identified that six of the seven included studies reported indicators of KDT efficacy in headache prophylaxis. Effects varied from reduced migraine frequency and intensity to migraine resolution [2,16,17,18,19,20,21]. However, the review highlighted limitations due to poor methodologies and weak study designs, including lack of control groups and small sample sizes. This confirms the need for RCTs in adequately powered studies to further investigate the impact of KDT for migraine treatment [2]. Additionally, other dietary modifications have been explored. A recent review of eight RCTs reported improvements in migraine attacks and/or severity following the use of a lower glycaemic index diet, low-fat diet, vegan diet, increased water intake and an elimination diet [22]. The most commonly listed foods identified as migraine triggers include caffeine, alcohol and foods containing amines and monosodium glutamate [22]. 

Given the lack of RCTs targeting migraine alleviation using KDT, the aim of the current pilot trial was to investigate whether KDT is superior to an evidence-informed healthy “anti-headache” dietary pattern (AHD) in improving migraine frequency, severity and duration.

## 2. Materials and Methods

### 2.1. Study Design

Total duration of the pilot randomised controlled crossover trial was 12 weeks, consisting of three phases: two dietary intervention periods (weeks 1–4 and 9–12) and one washout period (weeks 5–8), all of which were four weeks in duration. Participants were randomly assigned to receive either KDT or AHD first, followed by washout and the second intervention. This study was approved by the University of Newcastle Human Research Ethics Committee (Approval No. H-2017-0405). Written informed consent was obtained from all participants. The trial is registered with the Australian New Zealand Clinical Trials Registry (Trial No. 377161).

### 2.2. Participants and Recruitment

Participants were screened for eligibility via an online survey, using Qualtrics (qualtricsXM, Provo, UT, USA). Participants were eligible if they were aged 18 years and older, male or female, and from the Hunter and Central Coast region of New South Wales, Australia. Eligible participants were those with a history of migraines as defined in the International Classification of Headache Disorders (ICHD) II, Section 1 [23], and had experienced at least two episodes of moderate intensity headache (or greater) (determined by self-report), or at least five episodes of mildly intense headache in the previous four weeks, with ≥50% of headache days being migraine or probable migraine days. Participants were ineligible if they were following a specific medical nutrition therapy diet or had been diagnosed with any other neurological condition, as well as various medical conditions, such as history of gallbladder disease or pancreatic insufficiency. Eligibility criteria are outlined in Table 1.

Participants were recruited via flyers distributed across the University of Newcastle campus and via university email lists, posts on University social media (Facebook and Twitter) and a media release that targeted local radio and print media. These sources all contained links to the eligibility screener. If eligible, participants were contacted by a member of the research team. 

### 2.3. Intervention

Eligible participants were randomised to commence one of the two intervention groups: (1) AHD and (2) KDT. Participants attended baseline clinical assessment sessions consisting of two parts: (1) anthropometric and clinical data collection; and (2) a medical nutrition therapy consultation with a dietitian.

#### 2.3.1. Participant Medical Nutrition Therapy Consults

All participants received personal medical nutrition therapy consultations for a duration of 60–90 min with a dietitian. During the initial consultation, anthropometric outcomes were collected, and participants were prescribed either of the two dietary interventions and provided resources to assist with adherence to the diet regimes. Participants were also asked to download two smartphone applications (Migraine Buddy© app (Healint, Singapore) and Evernote (V6.11.2.7027, Evernote Corporation, Redwood City, CA, USA), described below), which they were instructed to use to log dietary intake and migraine data throughout the 4-week interventions. Participants were offered a $AU20 gift voucher at the initial consultation and at each follow-up measurement session to cover travel expenses.

#### 2.3.2. Ketogenic Diet Therapy

Three different KDT plans were developed in varying ratios of grams of total fat to carbohydrate plus protein (3:1, 2:1 and 1:1). Meal plans were devised by using the “keto diet calculator” [24] and adapted to match the macronutrient composition of foods found commonly in Australia. Meal plans were provided for a rotating seven-day, 1600–1800 kcal/day menu (females, 1600 kcal; males, 1800 kcal), presented as three meals per day. Participants had the option to snack on additional items from an approved list with a 3:1 ratio of total fat to combined carbohydrates plus protein, e.g., 30 g serve of nuts, or choose extra meals ad libitum from the meal plan to manage hunger and/or fatigue as needed. Participants were counselled by a dietitian to personalise their meal plans and optimise nutrient intakes and adherence. Participants were advised that they could flavour the meals with herbs, spices, salt and pepper, as desired, but should avoid using pre-packaged sauces to moderate total carbohydrate intake. They were instructed to abstain from alcohol and to not deviate from the meal plans. Black tea, coffee and artificially sweetened beverages, such as diet soft/soda drink, were recommended ad libitum. All participants started on a 3:1 diet plan and were monitored by a dietitian, with weekly follow-ups via email or telephone. If a participant indicated he/she was struggling to adhere to the initial 3:1 diet due to food portion sizes or was not tolerating the high-fat content or carbohydrate restriction, he/she was downgraded onto the 2:1 and then 1:1 in order to aid compliance and retention in the study, while achieving ketosis.

#### 2.3.3. Anti-Headache Diet

The AHD was based on current evidence for common dietary-related migraine triggers [25,26,27,28]. Participants were given a comprehensive list of foods and food items they were either allowed or that should be avoided. Common headache triggers, including caffeine, monosodium glutamate (MSG), sulphites, amines and histamines, were reduced or completely excluded, as were any reported individual dietary triggers. The diet promoted the consumption of raw or minimally processed foods (e.g., roasted nuts) rather than heavily processed items (e.g., commercially made microwave meal) and emphasised not skipping meals and adequate hydration (meeting fluid recommendations as recommended by the AGHE) [29]. There was no specific meal plan or calorie restriction; participants cultivated their own menus by using the provided information sheets as a guide.

#### 2.3.4. Washout Period

Between the two intervention diets, participants undertook a 2-week washout period and were instructed to resume their habitual dietary patterns without any dietary intervention. Participants were asked to continue recording entries in the Evernote and Migraine Buddy© during the “washout diet” period.

#### 2.3.5. Anthropometric Measurements

Anthropometric outcomes were measured at baseline and at four, eight and twelve weeks at the University of Newcastle, NSW, Australia. All measurements were performed by a trained researcher, using a standardised assessment protocol. Height (cm) was measured to 0.1 cm, without shoes, using the BSM370 Stadiometer (automatic) (InBody Co., Ltd., Seoul, Korea). The average of two measures was recorded. If the difference of the two measures was greater than 0.5 cm, a third measure was taken and an average of the closest two recorded. Weight (kg) was measured to 0.1 kg, using an InBody 770 composition analyser (InBody Co., Ltd., Seoul, Korea). Participants were advised to wear light clothing to each consult and removed any heavy items from their pockets. Two measures were recorded and the average value used. If the difference of the two measures was greater than 0.5 kg, a third measure was taken, and an average of the closest two was recorded. Body composition was also analysed by using the same InBody composition analyser to obtain total body fat percentage (BF%), skeletal muscle mass in kilograms (SMM) and fat mass in kilograms (FM).

#### 2.3.6. Migraine Buddy© App

The study intervention used the Migraine Buddy© app (Healint, Singapore), which tracked migraine frequency (number of migraine occasions), duration (hours and minutes) and severity (pain score out of 10), as well as migraine location, medications and relief methods used. Participants were asked to export their records to the research team via email. 

#### 2.3.7. Side Effects

Participants were provided with a symptom checklist and asked to tick off symptoms experienced on a given day, throughout the 4-week KDT. Symptom prompts included fatigue, constipation, stomach upset, bloating, flatulence, irritability, dizziness, difficulty sleeping and “other”.

#### 2.3.8. Food Box

At intervention commencement, participants were provided with a food box from a local supermarket, containing sample food products central to dietary adherence, and could be consumed regularly during each allocated dietary intervention period. The KDT food box included high-fat foods, such as butter and cream, as well as animal protein (chicken breast meat and beef mince) and low carbohydrate vegetables (baby spinach, green bell peppers and cucumber). The AHD food basket contained fresh fruit and vegetables, as well as herbal tea in which participants were advised to consume instead of caffeine containing tea and coffee. Each food basket was approximately $30 in value and contained enough food items for one week, following the prescribed dietary intervention.

#### 2.3.9. Physical Activity

Physical activity data were collected by using the International Physical Activity Questionnaire (IPAQ)—short form (7 questions) [30], which assesses physical activity across vigorous, moderate and walking activities. Any time variables exceeding 180 min per day were recoded to 180 min, in accordance with IPAQ recommendations. MET-minutes-per-week values were calculated for vigorous, moderate and walking physical activity and then summed to provide total MET-minutes per week. A categorical score was allocated to participants per the IPAQ scoring protocol as “High”, “Moderate” or “Low” physical activity.

#### 2.3.10. Quality of Life

Quality of life (QOL) was assessed pre and post each dietary intervention, using the Migraine Specific Quality of Life Questionnaire v2.1 (MSQ v2.1) [31]. The MSQ v2.1 has 14 questions assessing how migraine affected daily functioning over the past four weeks, across three domains: (1) Role Function Restrictive (RR), the degree to which performance of normal activities is limited by migraine; (2) Role Function Preventive (PR), the degree to which performance of normal activities is limited by migraine; and (3) Emotional Function (EF), emotional effects of migraine. Each response option is a 6-point Likert scale with responses ranging from “none of the time” to “all of the time”. Responses were assigned scores of 1–6, and scores computed as a sum of scores and scaled on a scale from 0 to 100, with higher scores relating to poorer QOL.

### 2.4. Diet Quality and Adherence to Interventions

#### 2.4.1. Australian Eating Survey

The Australian Eating Survey (AES), a 120-item validated food frequency questionnaire (FFQ), was administered for each pre- and post-intervention period [32]. Subjects were asked about frequency of consumption over the previous four weeks with frequency options ranging from “Never” up to “4 or more times per day” but varying depending on the food item. Data from the AES allowed us to calculate the total diet variety (scored out of a total of 73, using the Australian Recommended Food Score [ARFS]) [33], percentage of total energy intake from core and non-core foods (reflecting diet quality), and macronutrients and micronutrients (AUSNUT 2011-13 database). The ARFS, core vs. non-core food intake and micronutrient intake were used to evaluate baseline adequacy of dietary intake. Macronutrient intake data were used to assess adherence to the dietary interventions and evaluate whether dietary intake returned to baseline during the washout period. 

Participants undertaking KDT were asked to test their urinary ketone levels, using urinary regent sticks to track ketone body (acetoacetate) presence. A box of keto-diastix™ (Bayer Pharmaceuticals, Leverkusen, Germany) was provided to participants, along with written instructions on how and when to test urine samples. Participants were advised to test during each void until they achieved urinary ketosis and then test on waking and before each evening meal every second day. Participants recorded urinary ketone results, using a written diary, and returned it to the researcher at their follow-up session.

#### 2.4.2. Evernote App

During the initial consult, participants were asked to download the Evernote app onto their smartphone. The Evernote app (Evernote Corporation, Redwood City, CA, USA) allowed users to take photos of meals and snacks, write notes and place these items in a designated “notebook”. Each participant was set up with his/her own notebook which was linked to the research team’s central Evernote account. Participants were encouraged to collect images of all of their food and drinks for a minimum of three days per week, with one day being on the weekend. A researcher reviewed these images to assess participants’ adherence to their diet intervention and make contact with those who were not adhering.

### 2.5. Sample Size

A key objective of pilot studies is to gain initial estimates for a sample size calculation in a future adequately powered RCT, and thus a formal sample size calculation was not performed [8]. A systematic review of pilot and feasibility studies identified a median total sample size of approximately 30 in non-drug trials. Therefore, the recruitment target was set at 30 individuals [9].

### 2.6. Randomisation

The allocation sequence was generated by a computer-based random number generator. Participants were randomised to intervention order by a researcher who had no contact with participants during the trial. 

### 2.7. Outcomes

The primary outcome of this study was the difference in migraine frequency (number of migraine occasions), severity (pain level) and duration (hours and minutes) between diets. Each of these outcomes was recorded in the Migraine Buddy© app for all episodes of migraine throughout the study. Secondary outcomes included number of days to reach ketosis and frequency of symptoms whilst on KDT and change in body weight and composition and change in physical activity across both dietary interventions. 

### 2.8. Statistical Analyses

All statistical analyses were completed by using Stata data analysis and statistical software version 11.1 (Stata Corp., College Station, TX, USA) [34]. Baseline and change data are reported as either mean ± standard deviation (SD) for normally distributed variables or median (interquartile range (IQR)) for non-normally distributed variables. An independent t-test or non-parametric equivalent (rank sum) was used to determine any differences in demographic and dietary data between intervention groups at baseline. Wilcoxon rank rum was used to compare differences in migraine frequency, severity and duration between intervention groups. The level of significance was set at *p* < 0.05. As migraine data (frequency, duration and severity) were not collected at each of the four time-points (only collected if they occurred during the intervention), we were unable to perform any imputation or linear mixed models for an intension-to-treat analysis. Results presented are for those who completed all measurement sessions and the AES at each of the four time-points. For these completers, it was assumed that no migraine data meant no incidence of migraine during the intervention. 

## 3. Results

**Figure 1:**
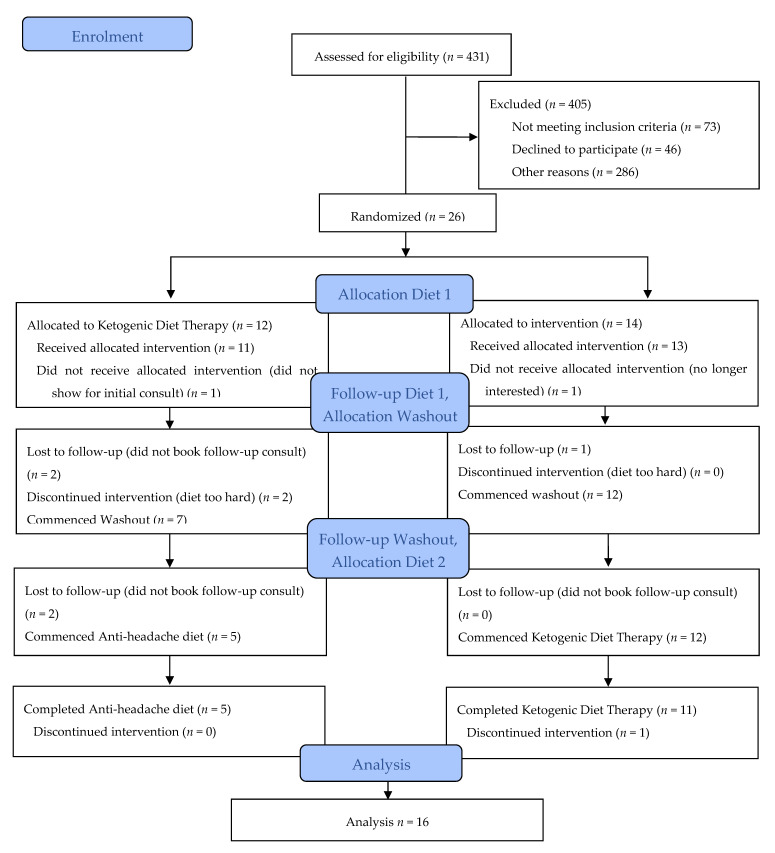
Consort diagram of participant flow.

Of the 431 people screened for eligibility 72 were eligible, of whom 26 (24 females and 2 males) provided informed consent and were randomised into either the AHD or the KDT (see Figure 1 consort flow diagram). Of these, two did not attend the baseline measurement session and were therefore excluded. A total of 16 participants completed all measurement sessions (33% dropout) and the AES at each of the four occasions and are therefore included in the final analysis. 

### 3.1. Baseline Characteristics

The baseline characteristics of the participants are summarised in Table 2. The mean age of completers was 42.6 ± 11.1 years, with 87.5% being female. Mean baseline weight was 77.5 ± 15.9 kg and, %BF was 35.6 ± 7.7. At baseline, five participants were classified as having a high physical activity level, nine moderate and two low physical activity levels. 

Supplementary Materials Table S1 summarises dietary intakes and comparisons to national recommendations for the whole sample at baseline. The mean total ARFS score was 35.4 ± 8.5 (out of max 73), which is classed as “getting there”, representing adequate, but not optimal, food variety. Alcohol intake made up 3.5 ± 3.6%, and core food intake made up 69.3 ± 14.8% of total energy intake, which is also reflected by adequate intake of most micronutrients. The two micronutrients that did not meet recommended intakes were folate and iron. Mean folate intake was 296 ± 103 μg (target 400 μg), and mean iron intake was 11.5 ± 3.8 mg (target 18 mg for females).

### 3.2. Food and Nutrient Intake at Baseline

At baseline, self-reported diet variety of the total completers sample was 35 out of a maximum of 73 points, and mean intake of core foods was 69.3% ± 14.8% (see Supplementary Materials Table S1). Out of 13 micronutrients assessed, 10 met the Recommended Dietary Intakes [35]. The three micronutrients that did not meet the RDIs were folate, iron and zinc (see Supplementary Materials Table S1). Macronutrient intake at baseline more closely aligned with Acceptable Macronutrient Distributions Ranges (AMDRs) [35]; however, mean carbohydrate intake was slightly below (41.6 ± 5.6% total energy (TE); AMDR 45–65%), and fat intake slightly higher (35.2 ± 5.3%TE; AMDR 20–35%) than the AMDRs (see Table 3). Saturated fat intake also exceeded the recommendation of <10% of TE (15.2 ± 3.5%).

### 3.3. Adherence

#### 3.3.1. Self-Reported Dietary Intake

There were no significant differences in total energy or macronutrient intakes between baseline and post-washout, thus suggesting that the washout period was successful (see Table 3). Following the two dietary interventions, %TE and grams per day from carbohydrate and fat were significantly different between KDT and AHD, with carbohydrate intakes higher on the AHD and fat intakes higher on KDT (see Table 3). Fat intake (g/day) during KDT was 1.5 times that of AHD (*p* < 0.01), whilst carbohydrate (g/day) was over half that of AHD (*p* < 0.001). Interestingly, self-report ratios of fat, carbohydrate + protein, did not reach that of the prescribed intervention whilst following KDT. However, the ratio during KDT was still three times higher than that of AHD (*p* < 0.01) (see Table 3).

#### 3.3.2. Urinary Ketones

A total of 13 (81%) participants measured urinary ketones across the 4-week KDT intervention. The average number of days where urinary ketones were recorded was 18 out of 28 (range 4–28 days), with the average ketone level being 7.2 mmol/L across the 28 days (range 2.0–14.0 mmol/L). The average number of days to reach ketosis was 2.2 ± 0.82 days. 

#### 3.3.3. Evernote

**Figure 2:**
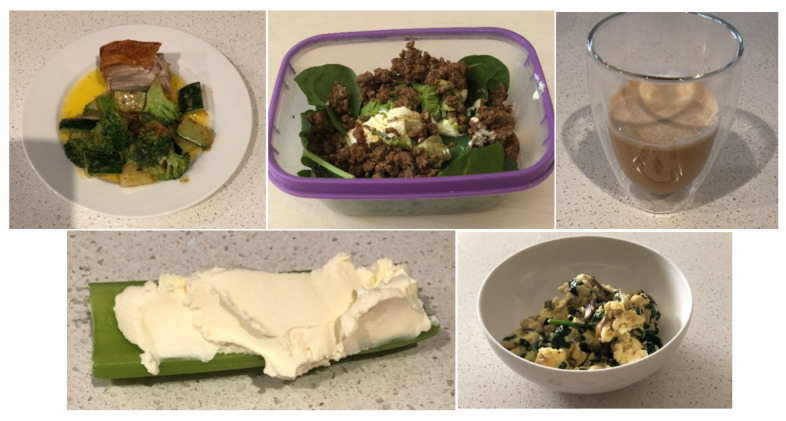
Example of one day of meals for a participant on a ketogenic diet.

Of the 16 participants with complete data, 14 kept an Evernote diary. On average, participants recorded images of their meals on an average of 24 days (range 13–51 days) across the two dietary interventions (8 weeks), averaging 3 days per week, which was in line with the recommendation. On those days, the number of meals captured averaged 2.5 (range 1.0–3.4) meals per day. Figure 2 illustrates examples of images recorded during KDT. 

### 3.4. Migraine Frequency, Severity and Duration

All 16 completers experienced migraine during AHD, with 14 of the 16 experiencing migraine during KDT. There were no statistically significant differences in migraine frequency, severity or duration (see Table 4) between dietary intervention groups. 

### 3.5. Changes in Weight, Physical Activity and Quality of Life 

Both groups had significant weight loss across the 4-week interventions (AHD −1.5 kg (−2.6, −0.4; *p* = 0.003); KDT −2.8 (−4.7, −1.5; *p* < 0.001), but there was no significant difference between groups (*p* = 0.080). No significant changes in physical activity were found within or between intervention groups. At baseline, the mean total quality-of-life score for the whole participant groups was 49.94 ± 14.15, and following each dietary intervention, the mean total quality-of-life scores were 49.13 ± 15.88 and 32.3± 11.58 for the AHD and KDT, respectively. The mean quality-of-life score for all three sub-scores and total QOL significantly improved on KDT but not AHD. During KDT, RR improved by 8.84 ± 6.58 (*p* = 0.0002), PR by 3.15 ± 2.62 (*p* = 0.0006), EF by 5.02 ± 4.07 (*p* = 0.0005) and total QOL by 17.0 ± 11.85 (*p* = 0.0001). Change in QOL was also significantly different between intervention groups (RR *p* = 0.007; PR *p* = 0.015; EF *p* = 0.029; total *p* = 0.006).

### 3.6. Side Effects during KDT

A total of 11 (69%) participants reported side effects during KDT, with the other five participants not completing the symptom checklist. The most frequently reported side effects were fatigue (*n* = 11 participants, average 8/28 days), stomach upset (*n* = 9 participants, 5/28 days) and constipation (*n* = 8 participants, 8/28 days). Participants following the KDT also reported difficulty sleeping (*n* = 7), dizziness (*n* = 5), irritability (*n* = 4), bloating (*n* = 2) and flatulence (*n* = 2). Participants experienced these symptoms, on average, 2 to 3 days out of 28 days. Of the 16 completers, four were stepped down to a 2:1 (*n* = 2) and 1:1 (*n* = 2) ratio. Those stepped down to 2:1 did so after five and eight days, and both that stepped down to 1:1 did so after four days following KDT. Only one of these four participants monitored their urinary ketones after the diet adjustment, with the record showing that they stayed in ketosis for the remainder of KDT. 

## 4. Discussion

Results from this crossover RCT comparing KDT to an AHD do not demonstrate the efficacy of KDT in reducing migraine frequency, severity and duration. However, while statistically insignificant, migraine duration on KDT was shorter by 6 h than AHD, which warrants further investigation into the impact of KDT on managing migraine frequency, severity and duration.

Findings from the current trial indicate that adults who experience migraine are unable to sufficiently alter their dietary intake in line with KDT prescriptions, and for those more closely aligning with prescriptions, following KDT is not without side effects. 

During the 4-week ketogenic diet intervention, over half of the participants reported side effects, including fatigue and gastrointestinal upset. This is in line with other research that reports frequent side effects associated with KDT and low adherence due to the restrictiveness of the diet [5,36]. This limits our capacity to investigate relationships between KDT and migraine outcomes, as well as our potential to explore this dietary approach further in longer-term studies. However, while side effects were frequently reported, an improvement in quality of life was seen. This suggests that, while side effects do occur during KDT, other improvements to migraine frequency, severity and duration, which were not able to be detected in this study, may occur that outweigh the reported side effects, improving overall quality of life. Additionally, the consistency of testing and reporting urinary ketones was poor within this study, which has important implications, as ketosis needs to be maintained in order to allow for the evaluation of whether there is an improvement in migraine outcomes directly related to KDT [17]. Future research should consider automated reminders, such a text-messages or emails, to measure and record ketone levels.

There are a number of recommended prescriptions for KDT, including the more intensive 4:1 ratio down to the more modest ratio of 1:1. In this study, self-reported dietary intake following KDT suggested that participants did not even reach a 1:1 ratio of fat: carbohydrate + protein. Four participants were stepped down to a 2:1 and 1:1 ratio, suggesting that adherence was difficult, and this could also explain the lower ratios reported for other participants whilst on KDT. Only one of the four stepped-down participants recorded urinary ketones, making it difficult to determine if ketosis remained even whilst consuming a lower ratio of fat: carbohydrate + protein. Given the difficulty with adherence and side effects reported, it would be worth investigating the lowest ratio that could be prescribed that still maintains physiological benefit. In this study, the highest reported urinary ketone level aligned with the deepest colour on the Keto Stix colour chart (daily average 14 mmol/L). This has previously been reported at the lower end of requirements for physiological benefit [8], so this suggests that, while most participants in this study did not reach a level of ketosis sufficient to reduce migraine frequency, severity and duration, effective KDT may not need to reach a ratio of 4:1. The authors also acknowledge that the use of urinary ketones does not quantify total ketone concentrations. Future research should consider the measurement of blood ketone concentrations via finger-prick analyses. 

While this study was unable to show any statistically significant differences in migraine frequency, severity and duration between the two dietary interventions, this is likely due to the small sample size. On the contrary, given the lack of significant difference between diets on migraine outcomes, it is also worth considering the quality of each diet in regard to nutrient intake and alignment with dietary guidelines. Following the AHD, macronutrient, saturated fat and fibre more closely aligned with national dietary guidelines compared to KDT. Therefore, in the longer-term, the AHD may be a more appropriate diet to follow for overall health, if it can be shown to have some benefit for migraine outcomes. Additionally, further research could consider trialing these dietary interventions with participants with higher headache frequency at baseline to determine whether a KDT is more beneficial in those with more frequent or severe migraine symptoms.

Migraine triggers vary from person to person, with common triggers including poor sleep patterns, stress, hormonal changes, bright lights and diet-related triggers, including caffeine, hypo-hydration and skipping meals [37,38]. The range of triggers adds further complexity for developing effective intervention strategies. Therefore, future research should consider how intervention strategies can be personalised to the triggers that are specific to individuals. 

### Limitations and Recommendations or Future Research

One of the main limitations of this study was the small sample size. While expressions of interest to participate in this study were above expectations, highlighting the prevalence of headache and interest in diet to resolve migraine, only a small number of participants met the eligibility criteria, and of those, 36% consented, with about one-third of those randomised not completing the full study protocol. Future research should expand inclusion criteria to also include those with severe headache, not migraine only. A further limitation was that baseline migraine characteristics cannot be reported, as this information was gathered in the eligibility screener before consent was obtained. Additionally, the sample was predominantly female and had factors such as the menstrual cycle potentially being one of the causes of migraine; thus, the findings cannot be transferred to the male population.

Another limitation of this study was that the dietary protocol prescribed did not take into account participant baseline intake and individual caloric requirements. While participants were allowed to eat ad libitum above the base protocol (only from a prescribed list of suitable ketogenic snacks), it would be beneficial for future studies to take a more individualised approach. One such example could be getting participants to keep a 14-day food record prior to commencing the intervention [39], or to estimate individual total daily energy requirements from equations and the ketogenic diet protocol to be based off individual caloric requirements. Future research should also consider strategies for reducing the rate of dropout through reducing the burden of following a KD and also monitoring. This could include less frequent measurement of urinary ketones than daily and commencing participants on a lower ketogenic ratio and only moving to a more restrictive ratio if ketosis is not reached. Data from this study suggest that this could be an appropriate strategy; however, there were insufficient data to make a strong recommendation. The inclusion of ketogenic meal-replacement supplements should also be considered due to the unpalatability of many ketogenic meal options. Lastly, this was only a short-term study, and not all participants experienced a migraine during this period. Future studies should consider trialing this diet for longer periods; however, further efforts to minimise participant burden and improve palatability of the diet would need to be addressed. 

## 5. Conclusions

Findings from the current trial indicate that adults who experience migraine are unable to sufficiently adhere to more restrictive KDT prescriptions, likely due to the associated side effects. Due to significant drop-out, this study was also underpowered to detect a difference in migraine outcomes between diets. However, given the clinically important difference in migraine duration (lower for KDT), further research in this area is warranted, with strategies implemented to lower participant burden and promote adherence and retention.

## Figures and Tables

**Table 1:** Participant eligibility criteria.

Inclusion Criteria	Exclusion Criteria
Any gender; Aged 18 and over; History of migraine headaches consisting of at least two episodes of moderate intensity headache (or greater), or at least five episodes of mildly intense headache in the previous four weeks, with ≥50% of headache days being migraine or probable migraine days.	Diagnosed with any other neurological condition (e.g., epilepsy or mental trauma); Medical condition requiring specific medical nutrition therapy (e.g., diabetes or coeliac disease); A history of strokes; Have a pacemaker; Are currently taking medications contraindicated whilst on KDT (e.g., blood pressure medication or insulin); Are pregnant or breastfeeding; Are not proficient in the English language; Have an intellectual or mental impairment where decision-making capacity is compromised; Do not own a smartphone; Have any of the following conditions: ➢ Gallbladder disease or removal of gallbladder, ➢ Had bariatric surgery, ➢ Pancreatic insufficiency, ➢ Liver disease, ➢ History of kidney stones,44 ➢ Eating disorder.

KDT, ketogenic diet therapy.

**Table 2:** Baseline characteristics of study participants enrolling in a dietary intervention for migraine prevention.

Characteristics	Data Are *n* (%) or Mean ± SD	
	Randomised	Completers
Participants (*n*)	24	16
Gender, female *n* (%)	22 (91.2)	14 (87.5)
Age (years)	42.6 ± 11.2	42.6 ± 11.2
Weight (kg)	79.4 ± 17.8	77.5 ± 15.9
Body fat (%)	38.4 ± 8.3	35.6 ± 7.7

**Table 3:** Dietary intake at baseline and during each 4-week phase for completers (*n* = 16) participating in a dietary intervention for migraine prevention.

	Baseline (*n* = 16)	Post-Washout (*n* = 16)	Post-Ketogenic (*n* = 16)	Post-Anti-Headache (*n* = 16)
	Mean ± SD		Mean ± SD	
Energy (kJ/day)	7894 ± 2740	7057 ± 2375	4979 ± 2738	4890 ± 2474
Carbohydrate (%TE)	42.6 ± 6.3	40.1 ± 6.8	14.5 ± 10.7	41.6 ± 9.0 *
Protein (%TE)	19.7 ± 3.0	21.1 ± 2.7	30.1 ± 6.8	23.4 ± 4.4 *
Fat (%TE)	34.0 ± 5.5	36.5 ± 5.0	54.6 ± 9.8	32.9 ± 7.0 *
Saturated fat (%TE)	14.6 ± 3.5	15.3 ± 3.7	21.9 ± 5.0	13.0 ± 4.3 *
Fat (g/day)	68.9 ± 23.4	65.7 ± 22.5	65.5 ± 20.9	41.8 ± 23.5 *
Carbohydrate (g/day)	199 ± 86	167 ± 68	53.8 ± 83.9	119.1 ± 60.4 **
Fibre (g/day)	25.3 ± 9.3	21.8 ± 6.6	14.9 ± 8.2	18.1 ± 8.1
Protein (g/day)	88.2 ± 25.2	86.6 ± 31.5	82.7 ± 31.4	64.2 ± 27.2
Mean ratio of Fat: Carbohydrate + Protein	0.3 ± 0.1	0.3± 0.1	0.6 ± 0.2	0.2 ± 0.1 *

Note: There were no significant differences in dietary intake between baseline and post-washout. TE = total energy. * Significant difference between dietary interventions (*p* < 0.01). ** Significant difference between dietary interventions (*p* < 0.001).

**Table 4:** Migraine frequency, duration and severity for each dietary intervention.

	Ketogenic (*n* = 16)	Anti-Headache (*n* = 16)	*p*-Value (Difference between Groups)
	Median [IQR]		
Migraine frequency (number of episodes over 4-week intervention)	3 [1, 7]	5 [2.5, 9]	0.205
Migraine duration (hours per episode)	11.2 [4, 21.4]	17.4 [9.4, 36.2]	0.152
Migraine severity (pain rating, 0–10, 10 being worst)	5.4 [3.5, 6.5]	5.5 [4.8, 6.2]	0.584

Note: There were no significant differences in migraine frequency, duration and severity between dietary interventions.

## Data Availability

The data presented in this study are available on request from the corresponding author. The data are not publicly available, due to ethical restrictions.

## Supplementary Materials

The following are available online at https://www.mdpi.com/article/10.3390/healthcare9091105/s1, Table S1: Baseline dietary intakes and national nutrient recommendations for the whole study sample.
