# Supplementary material for: Can Ketogenic Diet Therapy Improve Migraine Frequency, Severity and Duration?
Source: Healthcare (Basel). 2021 Aug 26;9(9):1105. doi: 10.3390/healthcare9091105 (PMC8471252; doi:10.3390/healthcare9091105)
Supplement: Supplementary file 1 [file healthcare-09-01105-s001.zip › healthcare-1296125-supplementary.pdf]

**Table S1**: Baseline dietary intakes and national nutrient recommendations for the whole study sample.

| | Baseline (*n* = 16) | Recommended intakes[#] |
|---|---|---|
| **Percentage of total energy intake from food groups** | | |
| ARFS* | 35.4 ± 8.5 | - |
| Core foods (%) | 69.3 ± 14.8 | - |
| Non-core foods (%) | 30.8 ± 14.8 | - |
| Alcohol (%) | 3.5 ± 3.6 | - |
| Water (L) | 3.1 ± 0.9 | - |
| **Micronutrients** | | |
| Thiamin (mg) | 1.3 ± 0.5 | 1.1mg/d |
| Riboflavin (mg) | 1.7 ± 0.7 | 1.1mg/d |
| Niacin (mg) | 20.3 ± 7.0 | 14mg/d |
| Vitamin C (mg) | 176 ± 74.0 | 45mg/d |
| Folate (μg) | 296 ± 103 | 400μg/d |
| Vitamin A (μg) | 1361 ± 537 | 700μg/d |
| Sodium (mg) | 2046 ± 746 | 2000mg/d |
| Potassium (mg) | 3167 ± 1068 | 2800mg/d¦ |
| Magnesium (mg) | 357 ± 105 | 320mg/d |
| Calcium (mg) | 1008 ± 367 | 1000mg/d |
| Phosphorus (mg) | 1443 ± 451 | 1000mg/d |
| Iron (mg) | 11.5 ± 3.8 | 18mg/d |
| Zinc (mg) | 12.0 ± 3.3 | 8mg/d |

*ARFS: Australian Recommended Food Score (max points 73); #Recommended intakes: Value provided is the Recommended Dietary Intake for adult females aged 31-50yrs; ¦Adequate intake.
